# DFT Study of the Structure, Reactivity, Natural Bond Orbital and Hyperpolarizability of Thiazole Azo Dyes

**DOI:** 10.3390/ijms18020239

**Published:** 2017-02-01

**Authors:** Osman I. Osman

**Affiliations:** 1 Chemistry Department, Faculty of Science, P.O. Box 80203, Jeddah 21589, Saudi Arabia; oabdelkarim@kau.edu.sa; Tel.: +966-126-951-795; 2 Chemistry Department, Faculty of Science, University of Khartoum, P.O. Box 321, Khartoum 11111, Sudan

**Keywords:** thiazole azo dyes, donor–acceptor, HOMO-LUMO, UV–Vis., NLO, NBO

## Abstract

The structure, reactivity, natural bond orbital (NBO), linear and nonlinear optical (NLO) properties of three thiazole azo dyes (A, B and C) were monitored by applying B3LYP, CAM-B3LYP and ωB97XD functionals with 6-311++G** and aug-cc-pvdz basis sets. The geometrical parameters, dipole moments, HOMO-LUMO (highest occupied molecular orbital, lowest unoccupied molecular orbital) energy gaps, absorption wavelengths and total hyperpolarizabilities were investigated in carbon tetrachloride (CCl_4_) chloroform (CHCl_3_), dichloromethane (CH_2_Cl_2_) and dimethlysulphoxide (DMSO). The donor methoxyphenyl group deviates from planarity with the thiazole azo moiety by ca. 38°; while the acceptor dicyanovinyl, indandione and dicyanovinylindanone groups diverge by ca. 6°. The HOMOs for the three dyes are identical. They spread over the methoxyphenyl donor moiety, the thiazole and benzene rings as π-bonding orbitals. The LUMOs are shaped up by the nature of the acceptor moieties. The LUMOs of the A, B and C dyes extend over the indandione, malononitrile and dicyanovinylindanone acceptor moieties, respectively, as π-antibonding orbitals. The HOMO-LUMO splittings showed that Dye C is much more reactive than dyes A and B. Compared to dyes A and B, Dye C yielded a longer maximum absorption wavelength because of the stabilization of its LUMOs relative to those of the other two. The three dyes show solvatochromism accompanied by significant increases in hyperpolarizability. The enhancement of the total hyperpolarizability of C compared to those of A and B is due to the cumulative action of the long π-conjugation of the indanone ring and the stronger electron-withdrawing ability of the dicyanovinyl moiety that form the dicyanovinylindanone acceptor group. These findings are facilitated by a natural bond orbital (NBO) technique. The very high total hyperpolarizabilities of the three dyes define their potent nonlinear optical (NLO) behaviour.

## 1. Introduction

In recent decades, researchers have become interested in the fabrication of metal surfaces that are functionalized with organic chromophores for tailoring their electrical, magnetic, optical and electroptical properties [1,2,3,4,5]. The adjustment of their key surface properties could be achieved by proper molecular design and/or by fine control of their film structure at the molecular level [6]. The proper chromophores suitable for these criteria could occur as organic second-order nonlinear optical (NLO) compounds having an electron donor (D) and electron acceptor (A) separated by a π-conjugated spacer (D-π-A) [7]. These structural rearrangements facilitate asymmetrical ground-state charge transfer emanating from the donor group (D) through the π-linker to the acceptor moiety (A) under the influence of an electric field [8]. The last few decades have witnessed the fabrication of thermally and photochemically stable NLO benzenoid chromophores with potent hyperpolarizabilities [9,10,11]. Toward the end of last century, a series of single-substituted thiazole ring and donor–acceptor thiazole-containing chromophores were synthesized, characterized and their superior hyperpolarizabilities compared to oxazoles, imadazoles and thiophenes were obtained [12,13]. In 2004, semi-empirical and ab initio calculations were performed on a series of push-pull π-conjugated styryl benzothiazoles dyes [14]. At the end of last decade, benzothiazolium salts having dimethylamino and diphenylamino electron-donating and nitro or cyano electron-withdrawing groups were synthesized and studied for their NLO properties both theoretically and experimentally by using Hyper-Rayleigh scattering [15]. In 2011, the linear and nonlinear properties of a series of triphenylamine-derived benzothiazoles were tuned by incorporating some electron-withdrawing groups [16]. Extremely polarizable NLO chromophores were manufactured also by incorporating the five-membered heteroaromatic thiazole, pyrrole or thiophene rings [17,18,19] and/or thiazole-annulated heteroaromatics, such as benzobisthiazole [20]. It has been established theoretically and experimentally that the location of the heteroatoms within heteroaromatic rings and the relative position of electron-donor and electron-acceptor groups could play vital roles in dictating the NLO activity of these chromophores [14,15,16,20,21,22,23].

As colorants, azo dyes are effectively used in the textile, leather and paints industries [24]. In particular, thiazole azo dyes with vivid colors, especially reds, oranges and yellows [25,26,27,28], have substantial bathochromic absorptions relative to their benzenoid peers as a result of having more electronegative heteroatoms that act as auxiliary electron acceptors [22,23,29,30].

El-Shishtawy et al. [31] have synthesized and investigated, experimentally, the structure and NLO properties of three thiazole azo dyes with a lateral methoxyphenyl donor group coupled with indandione, malononitrile and dicyanovinylindanone acceptor moieties. They showed that the three dyes are thermally stable and have strong absorption wavelengths whose maxima are dictated by the nature of the acceptor groups. In addition, they have also demonstrated that the dye with the dicyanovinylindanone acceptor group is an extremely promising NLO devise with a nonlinearity μβ coefficient amounting to three times those of the other two dyes. In this paper, we endeavor to further complement their findings theoretically and computationally. Density functional theory (DFT) and Time-dependent density functional theory (TD-DFT) with traditional hybrid and long-range corrected (LC) functionals and moderate basis sets will be used to monitor their photochromic and NLO properties. The intramolecular charge transfer (ICT) of the three dyes, from the methoxyphenyl donor toward the three-acceptor groups, will be studied by natural bond orbital (NBO) technique.

## 2. Results and Discussion

### 2.1. Geometrical Analysis

**Figure 1:**
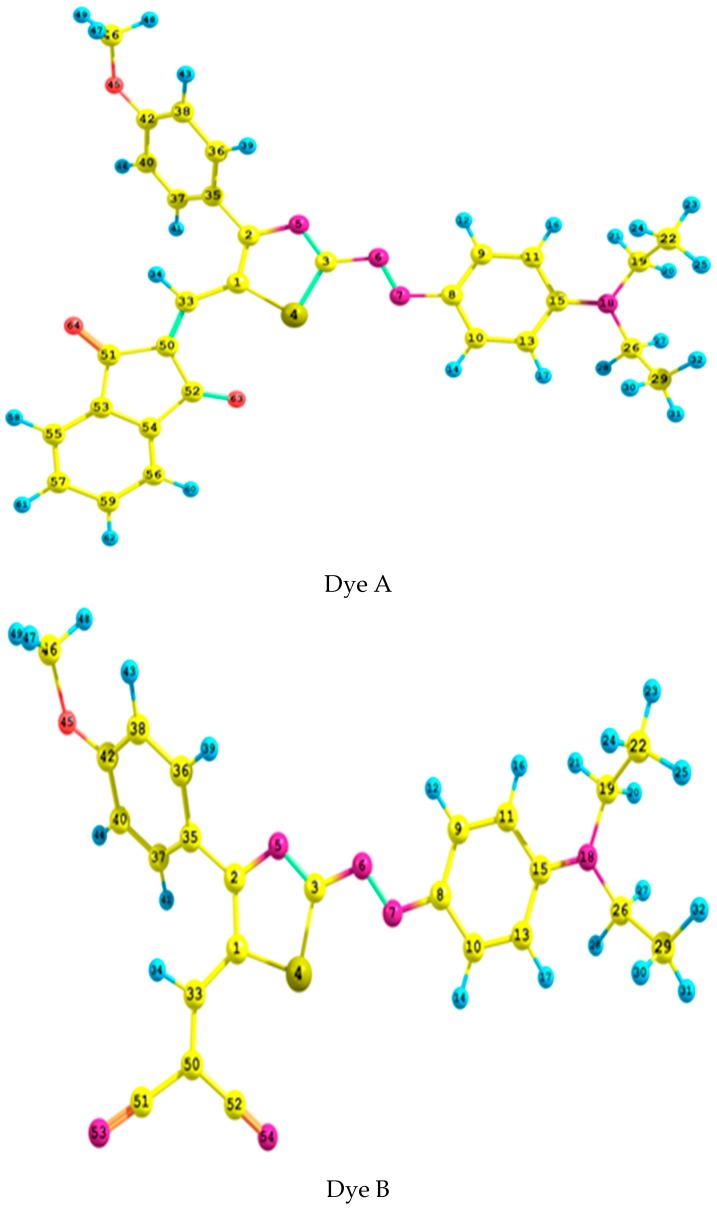

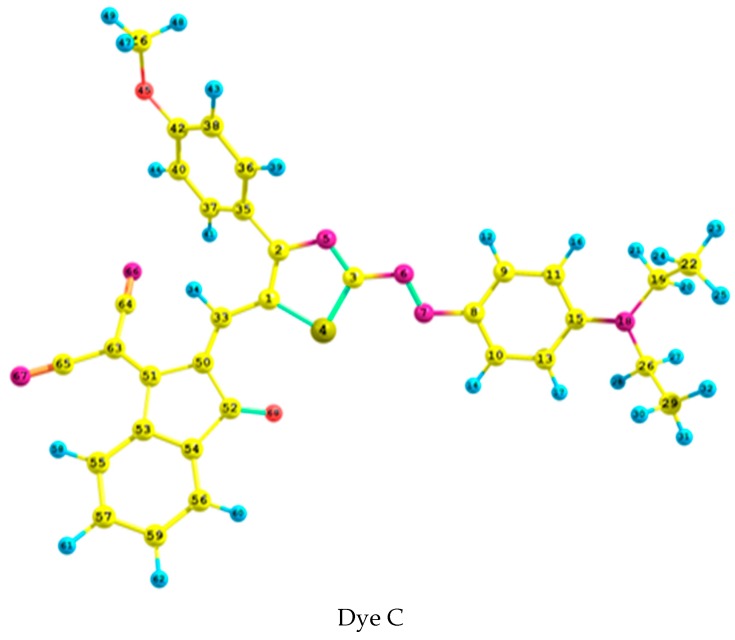
An atom numbering scheme of Dyes A, B and C. The carbon, nitrogen, oxygen, sulphur and hydrogen atoms are indicated by yellow, pink, red, orange and blue colours, respectively.

The structure of the three dyes A, B and C were optimized to global minima using B3LYP, CAM-B3LYP and ωB97XD functionals with aug-cc-pvdz and 6-311++G** basis sets (See Figure 1). Some selected bond lengths and dihedral angles of these optimization procedures together with those extracted from the crystal structures of dyes B and C [31] were listed in Table 1. In excellent agreement with the available crystallographic data [31], all the geometry optimizations of the dyes indicated that the donor methoxyphenyl group deviates from planarity with the thiazole azo moiety by ca. 38°; while the acceptor dicyanovinyl and dicyanovinylindanone groups diverge by ca. 6°. As Table 2 shows, an overall excellent agreement between the measured [31] and calculated values is met. This is indicated by the small absolute errors. These findings agree satisfactorily with similar theoretical computations [32]. The N6–N7 crystallographic [31] and calculated bond lengths of dyes B and C are in overall good agreement with each other, having average errors maxima of 0.031 and 0.038 Å, respectively. Dye B crystallographic S4-C1 bond length of 1.744 Å was exactly reproduced by ωB97XD/6-311++G** level of theory but with an error of 0.012 Å in dye C. Generally, the bond lengths computed by the different DFT functionals with the aug-cc-pvdz basis set are slightly longer and much closer to the crystallographic values [31] compared to those estimated by the 6-311++G** basis set. The multiple bond character of C1–C33 bond with an average value of 1.426 Å is indicated by being shorter than that of 2-methylthiophene [33] by ca. 0.079 Å. That is, C1 and C33 are nearly sp^2^ hybridized. The sp^2^ hybridization environment around the bridge between the acceptor groups and the thiazole azo moiety is also facilitated by the value of the angle C33–C1–C2 being ca. 124°. An unexpected agreement between the crystallographic [31] and the computed dihedral angles occurred satisfactorily, although the two measurements were carried out in different phases [34].

### 2.2. Frontier Molecular Orbitals (FMOs)

**Figure 2:**
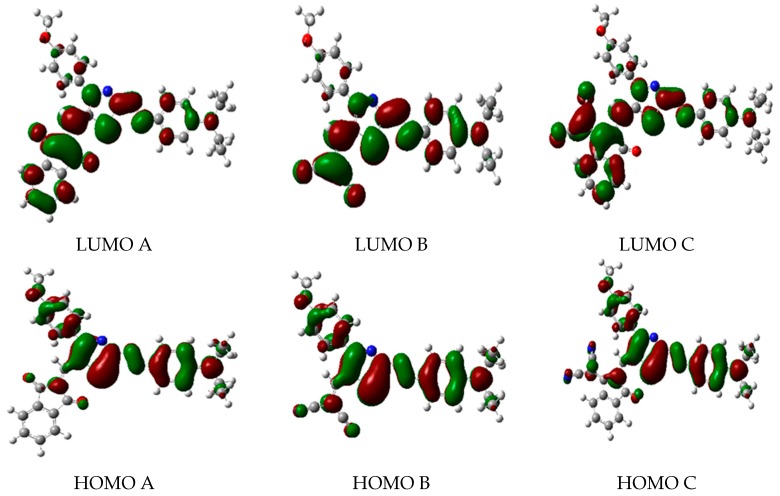
The highest occupied molecular orbitals (HOMOs) and the lowest unoccupied molecular orbitals (LUMOs) of the three dyes, A, B and C, which have been simulated by using HF/3-21G//B3LYP/6-311++G** level of theory. The orbital wave functions are positive in the red regions and negative in the green.

The frontier molecular orbitals (FMOs) are formed mainly by the highest occupied molecular orbital (HOMO) and the lowest unoccupied molecular orbital (LUMO). The FMOs participate strongly in investigating the electrical and chemical properties of substrates [35]. They affect these properties through forming their polarities, together with their abilities for absorbing light. This means that they act as donor and acceptor orbitals [36], respectively. The HOMO and LUMO orbitals, the chemical hardness (η), the electronic chemical potential (μ) and the global electrophilicity index (ω) using the elected DFT functionals and basis sets are listed in Table 3. Figure 2 depicts the HOMO and LUMO orbitals for the studied dyes (A, B and C). On the one hand, the HOMOs for the three dyes are identical. They spread over the methoxyphenyl donor moiety, the thiazole and the benzene rings as π-bonding orbitals. On the other hand, the nature of the acceptor moieties dictated the energy and the shape of the LUMOs. The LUMOs of the A, B and C dyes extend over the indandione, malononitrile and dicyanovinylindanone acceptor moieties, respectively, as π-antibonding orbitals. The strengths of these acceptor groups are reflected in the stabilization of the LUMOs [31] in excellent agreement with our results shown in Table 3. The kinetic stability of the three dyes can be monitored by the HOMO-LUMO energy gaps [37]. This means that smaller HOMO-LUMO splittings lead to lower kinetic stability and higher chemical reactivity. The reverse is equally true. In addition, it is energetically favorable to move electrons easily between high-lying HOMOs and low-lying LUMOs [38]. All our results showed that Dye C is much more reactive than dyes A and B. However, dyes A and B are of comparable reactivity. With the exception of ωB97XD/6-311++G** model chemistry, all other elected levels of theory revealed that Dye A is slightly more reactive than Dye B.

Likewise, the chemical hardness (η) is useful in studying the stability and reactivity of compounds. It is formulated in terms of the energies of the HOMOs and LUMOs [40]:

(1) $$\eta =\left(\frac{E_{HOMO}-E_{LUMO}}{2}\right)$$

This formula indicates that soft compounds have small chemical hardness, while hard ones have large chemical splittings. In other words, soft compounds have small excitation energies, that is, their electron densities are easily altered, while hard ones have large excitation energies or their electronic densities are difficult to modify [40]. Referring back to Table 3, it can be seen that Dye C is the softest, while Dye B is the hardest, among the three dyes. 

Moreover, the electronic chemical potential (µ) shows the escaping tendency of electrons in compounds [41] and given by Equation (2) [42,43]:

(2) $$\mu =-\left(\frac{E_{HOMO}+E_{LUMO}}{2}\right)$$

As Table 3 shows, the order of the electronic chemical potential for the three dyes is as follows: B ˃ C ˃ A. The global electrophilicity index (ω) estimates the stabilizing energy when a surrounding environment supplies a chemical entity with an additional electronic charge. The index (ω) relates to the electronic chemical potential (µ) and the chemical hardness (η) through Equation (3) [41]:

(3) $$\omega =\frac{\mu^{2}}{2\eta }$$

The values of the global electrophilicity indexes shown in Table 3 for the three dyes indicate that Dye A is the strongest nucleophile, while Dye C is the strongest electrophile among the three substrates.

### 2.3. UV–Visible Spectral Analysis

It has become general knowledge that the π→π* and n→π* electronic transitions in π-conjugated organic compounds lead to UV–Vis. spectra [44]. They are due to electron motions between the FMOs; like the promotion of an electron from the HOMO to the LUMO. Dyes A, B and C have many π-bonds in the thiazole azo, the two benzene and indane rings, together with the nitrogen, oxygen and sulphur atoms lone pairs. The experimental and theoretical maximum absorption wavelength (λ_max_) for dyes A, B and C in chloroform are depicted in Table 4. The estimated values are computed by the time-dependent density functional theory (TD-DFT) [45] procedure using the polarizable continuum model (PCM) method [46] with TD-B3LYP/6-311++G** model chemistry. The experimental maximum wavelength bands for A, B and C dyes in chloroform showed up at 623, 619 and 687 nm, respectively [31]. The longest wavelength due to Dye C is ca. 10% longer than the average wavelength of the other two dyes. This difference in trend was nearly reproduced theoretically using the elected levels of theory. That is, our calculated maximum wavelengths, in chloroform, of Dye C are longer than those of dyes A and B by 5%–12%. In excellent agreement with El-Shishtawy et al. [31], the longer maximum wavelength of Dye C resulted from its more stabilized LUMO relative to the LUMOs of dyes A and B. It can be concluded that the extra stability of the LUMO of Dye C (see Table 3) originates from the stronger electron-withdrawing ability of the dicyanovinylindanone group compared to those of the indandione and dicyanovinyl moieties [31]. The UV–Vis. spectra of the three dyes (A, B and C) are simulated in CCl_4_, CHCl_3_, CH_2_Cl_2_ and DMSO solvents applying TD-CAM-B3LYP/6-311++G** level of theory. The results are shown in Table 5. The interactions between these solvents and the dyes render them fairly solvatochromic, with red shifts of 0.063, 0.065 and 0.039 eV in the π→π* bands of A, B and C, respectively, on moving from the less polar CCl_4_ to the highly polar DMSO. As Table 5 shows, the HOMOs and LUMOs of the dyes are further stabilized relative to the polarity of the solvents, with the latter being greatly affected. It seems, then, that the solvatochromic behaviour could have resulted mainly from the relatively strong interactions between these solvents and the indandione, malononitrile and dicyanovinylindanone acceptor moieties [47].

### 2.4. Nonlinear Optical (NLO) Properties

Experimentalists and theoreticians have adopted many different methods and conventions for the determination of hyperpolarizability. This situation has brought about some form of ambiguity when theoretical data are compared with measured ones [48]. In this contribution, we compute the total hyperpolarizability, β_tot_, by the relation:

(4) $$\beta_{\mathrm{tot}}=\sqrt{\left(\beta_{x}^{2}+\beta_{y}^{2}+\beta_{z}^{2}\right)}$$

where

(5) $$\beta_{i}=\beta_{iii}+\frac{1}{3}\sum \left(\beta_{ijj}+\beta_{jij}+\beta_{jji}\right)$$

The total hyperpolarizabilities in atomic units (a.u.) are related to the electrostatic units (esu) by the relation: 1 a.u. = 8.6393 × 10^−33^ esu.

The computed dipole moments, energy gaps and the total hyperpolarizabilities for gas-phase dyes A, B and C, together with those of gas-phase p-nitroaniline (pNA) using B3LYP/6-311++G** model chemistry, are listed in Table 3. pNA is selected for comparison purposes because it is a typical example of a donor–acceptor charge-transfer species with high hyperpolarizability that was exposed to both experimental [39] and theoretical [49] investigations. In Table 3, it can be easily seen that dyes A and B have nearly equal total hyperpolarizabilities, which amount to 14- and 12-fold that of pNA, respectively, using the same level of theory. In contrast, the total hyperpolarizabilities of Dye C amount to twice those of dyes A and B and up to ca. 25-fold that of pNA. In Table 4 are listed the estimated dipole moments, energy gaps and total hyperpolarizabilities of the chloroform-solvated dyes (A, B and C) using the elected levels of theory together with their experimental counterparts in the same solvent. Apparently, the B3LYP functional overestimated the total hyperpolarizabilities of both the gas-phase and solution substrates compared to those obtained from CAM-B3LYP and ωB97XD counterparts, while the latter functionals yielded comparable values [49]. In addition, the total hyperpolarizabilities of the solvated dyes are ca. three–fold greater than their gas-phase peers [50]. This phenomenon could be related to the increase of dipole moment change between the ground and excited states [15] (cf. Table 3 and Table 4). Our calculated total hyperpolarizabilities of the three dyes in chloroform are in good match with the measured ones in the same solvent [31]. In addition, the estimated total hyperpolarizabilities of the three dyes in CCl_4_, CHCl_3_, CH_2_Cl_2_ and DMSO solvents using CAM-B3LYP/6-311++G** level of theory, listed in Table 5, are dictated by the polarity of these solvents, that is, the solvatochromic behaviours of the dyes have brought about pronounced NLO characteristics [47]. As Table 3, Table 4 and Table 5 show, the values of total hyperpolarizabilities of the three dyes (A, B and C) are inversely proportional to the magnitude of the energy gaps [51,52,53]. This is because narrow energy gaps enhance intramolecular charge transfer and hence produce larger total hyperpolarizabilities. In addition, the total hyperpolarizability of Dye A is slightly more than that of Dye B, in spite of the fact that the dipole moment of the latter is ca. twice that of the former. The enhanced total hyperpolarizability of dyes A and C could be due to the longer extension of the π-conjugation of the indandione and dicyanovinylindanone moieties compared to that of the dicyanovinyl group, while Dye B competes with them through the stronger electron-withdrawing ability of the dicyanovinyl moiety [31].

### 2.5. Natural Bond Orbital (NBO) Analysis

The second-order perturbation energies ($E^{\left(2\right)}$) are commonly used as a quantitative tool for investigating bonding and antibonding interactions through natural bond orbital (NBO) technique [54,55,56,57]. The second-order perturbation energies ($E^{\left(2\right)}$) are given by Equation (6):

(6) $$E^{\left(2\right)}=q_{i}\frac{F^{2}\left(i,j\right)}{\varepsilon_{j}-\varepsilon_{i}}$$

where the term $F^{2}\left(i,j\right)$ represents the off-diagonal matrix elements, $q_{i}$ gives the donor orbital occupancy, $\varepsilon_{i}$ and $\varepsilon_{j}$ estimate the donor and acceptor orbital energies, respectively. The quantities of $E^{\left(2\right)}$ evaluate the magnitude of interaction between the donor and acceptor orbitals. They, therefore, show the extent of delocalization throughout the chemical species. Table 6 lists the extremely influential interactions between the bonding or lone pair Lewis Type NBO occupied orbitals with antibonding Non-Lewis NBO unoccupied orbitals of dyes A, B and C. They were estimated by using HF/6-31+G*//B3LYP/6311++G** level of theory. The n→π* and π→π* interactions define the charge transfer from the donor methoxyphenyl group towards the acceptor moieties and contribute effectively to the stabilization of the three dyes. The former interactions (n_2O45_→π*_C38–C42_) stabilized A, B and C with 44.70, 45.65 and 44.95 kcal/mol, respectively, while the latters, inclusively, availed 220.95, 211.81 and 287.83 kcal/mol, respectively, for their stability. The most effective of these interactions is the movement of charge through the phenyl ring (π_C38–C42_→π*_C35–C36_) that stabilized A, B and C by 58.90, 54.83 and 55.10 kcal/mol, respectively. The charge transfer from the methoxyphenyl ring towards the Thiazole ring (π_C35–C36_→π*_C1–C2_) contributed 23.31, 23.69 and 22.83 kcal/mol for the stability of dyes A, B and C, respectively. The π_C1–C2_→π*_C35–C50_ interactions indicate the flow of charge from the Thiazole ring towards the acceptor moieties. They avail 40.80, 43.29 and 49.30 kcal/mol for the stabilization of A, B and C, respectively. It is interesting to note that those four π→π* interactions, which involve similar paths, benefited the three dyes by comparable amounts of delocalization energies. In addition, it is noteworthy to mention that both A and B dyes are devoid of the movement of charge from the Thiazole ring towards the dicyanovinyl moiety via the indanone ring (π_C33–C50_ →π*_C51–C63_) which signified Dye C by 37.99 kcal/mol. As a result, Dye C is characterized by huge hyperconjugative energy compared to A and B, while the latters have comparable delocalization energies. These comparable hyperconjugative interactions included π_C33–C50_→π*_C51–O64_ and π_C33–C50_ →π*_C52–O63_ delocalizations, which stabilized Dye A by 34.05 and 33.30 kcal/mol, respectively, while the π_C33–C50_→π*_C51–N53_ and π_C33–C50_→π*_C52-N54_ interactions subserved Dye B by 30.50 and 28.67 kcal/mol, respectively.

The huge charge transfer that occurred in Dye C explains its larger total hyperpolarizability compared to those of A and B. On the one hand, this is because Dye C has both the long π-conjugation extension of the indanone moiety and the strong electron-withdrawing ability of the dicyanovinyl group. On the other hand, dyes A and B have either the long π-conjugation or the strong electron-withdrawing potency. The large dipole moment and the strong electron-withdrawing ability of Dye B yielded total hyperpolarizabilities comparable to those of Dye A that has a smaller dipole moment. The competitiveness of Dye A originates from the long π-conjugation extension associated with the indandione ring [31]. The intramolecular charge transfer associated with the π_C1–C2_→π*_C35–C50_ interaction is effectively shown by the C1–C33 bonds being multiply bonded (1.419–1.431 Å). All these findings are in excellent agreement with those reported by El-Shishtawy et al. [31].

## 3. Computational Details

The Gaussian09 Suite of programs [58] were used to perform the quantum mechanical molecular orbital calculations of the three dyes, A, B and C. The GaussView [59] and Chemcraft [60] softwares were applied to monitor the structure and properties of the studied dyes. A number of density functional theory (DFT) [61] functionals with the triple zeta and polarization functions at the hydrogen and carbon atoms (6-311++G**) [62,63,64] and augmented correlation-consistent polarization with double zeta functions (aug-cc-pvdz) [65] basis sets were tested. They were used to optimize the geometrical structures of those dyes for investigating their reactivity, hyperpolarizability, linear and nonlinear optical (NLO) properties. The DFT functionals comprise the Becke, three-parameter, Lee–Yang–Parr exchange–correlation hybrid functional (B3LYP) [66,67], the Coulomb-attenuating method that includes the hybrid features of B3LYP and the long-range correction (CAM-B3LYP) [68], and the long-range corrected (LC) hybrid density functional with empirical atom–atom dispersion corrections (ωB97XD) [69]. The Gaussian NBO software [56,70] was applied to perform a natural bond orbital (NBO) analysis for the three dyes.

The time-dependent density functional theory (TD-DFT) [71] and the polarizable continuum model (PCM) [72] technique were applied to compute the UV–Vis. spectra of the CCl_4_, CHCl_3_, CH_2_Cl_2_ and DMSO solvated dyes using the elected levels of theory. The frontier molecular orbitals (FMO) and the global chemical reactivity descriptors of the three dyes were examined by using all tested model chemistries

## 4. Conclusions

The geometry, reactivity, linear and nonlinear optical behaviour of three donor–acceptor thiazole azo dyes were monitored by DFT calculations. The donor methoxyphenyl group and the acceptor indandione, malononitrile and dicyanovinylindanone moieties, incorporated into dyes A, B and C, respectively, were not coplanar with the thiazole azo spacer group. The HOMO-LUMO analysis showed that Dye C is more reactive than both A and B. This property ties up nicely with the longer absorption wavelength of the former. This linear behaviour is assigned to both the long π-conjugation extension of the indanone ring and the strong electron-withdrawing ability of the dicyanovinyl moiety. The three dyes showed solvatochromism on moving from CCl_4_ to DMSO solvents. The solvatochromic behaviours were reflected in pronounced NLO properties. The calculated total hyperpolarizabilities of Dye C were more than two-fold those obtained from A and B and ca. 25-fold of that from pNA. An NBO investigation supported these results. The enhancement of the linear and nonlinear behaviour of Dye C originates, in part, from the π_C33–C50_→π*_C51–C63_ transition, which stabilized this dye by 37.99 kcal/mol. The delocalization energies of A and B are nearly comparable, with the former having a slightly higher amount. This is because the latter is devoid of the π_C33–C50_→π*_C51–O64_ and π_C33–C50_→π*_C52–O63_ transitions, which stabilized the former by 67.35 kcal/mol, while the former is destitute of the π_C33–C50_→π*_C51–N53_ and π_C33–C50_→π*_C52–N54_ transitions, which availed 59.17 kcal/mol for the latter. All our theoretical findings are in excellent agreement with experiment [31] and affirm the use of the three dyes as potential NLO devices.

## Figures and Tables

**Table 1:** Some selected bond lengths (Å) and dihedral angles (degrees) of the optimized structures of A (top 6 lines), B (line 7 to 13) and C (bottom 7 lines) dyes which have been estimated by using different DFT functionals with 6-311++G** and aug-cc-pvdz basis sets. The crystal structures for B and C dyes are listed for comparison purposes.

Parameter		N6–N7	N5–C2	N5–C3	C1–C2	C33–C50	S4–C1	C1–C33	C33–C1–C2	C15–N18–C26–C29	C50–C33–C1–C2	C41–C40–C2–N5
B3LYP	6-311++G**	1.269	1.357	1.314	1.412	1.366	1.760			−82.8		−36.3
	Aug-cc-pvdz	1.274	1.360	1.318	1.416	1.372	1.766	1.423	124.3	−83.0	176.5	−35.9
CAM-B3LYP	6-311++G**	1.251	1.358	1.302	1.394	1.352	1.747	1.427	123.8	−81.7	175.7	−37.8
	Aug-cc-pvdz	1.256	1.361	1.306	1.399	1.358	1.753	1.429	124.0	−81.9	176.0	−37.4
ωB97XD	6-311++G**	1.252	1.359	1.303	1.395	1.353	1.745	1.429	123.8	−78.2	173.6	−38.1
	Aug-cc-pvdz	1.257	1.362	1.307	1.399	1.358	1.751	1.431	123.9	−77.4	173.9	−37.9
B3LYP	6-311++G**	1.272	1.358	1.311	1.408	1.374	1.759	1.419	125.0	−83.2	176.0	37.0
	Aug-cc-pvdz	1.277	1.362	1.316	1.412	1.378	1.764	1.422	125.1	−83.1	176.4	36.5
CAM-B3LYP	6-311++G**	1.254	1.359	1.300	1.391	1.358	1.746	1.424	124.7	−81.3	174.8	38.6
	Aug-cc-pvdz	1.259	1.362	1.304	1.396	1.363	1.752	1.427	124.8	−81.3	175.4	38.1
ωB97XD	6-311++G**	1.254	1.360	1.301	1.392	1.359	1.744	1.428	124.4	−76.0	173.7	39.6
	Aug-cc-pvdz	1.259	1.363	1.306	1.396	1.364	1.750	1.430	124.6	−76.0	174.2	39.3
Expert. ^1^		1.297	1.368	1.318	1.382	1.361	1.744	-	-	−88.8	-	34.6
B3LYP	6-311++G**	1.272	1.353	1.316	1.423	1.381	1.767	1.419	123.6	−82.3	170.6	−39.3
	Aug-cc-pvdz	1.276	1.356	1.320	1.427	1.387	1.773	1.421	123.7	−82.4	171.3	−37.8
CAM-B3LYP	6-311++G**	1.253	1.354	1.303	1.403	1.365	1.753	1.426	123.1	−81.2	170.9	−41.6
	Aug-cc-pvdz	-	-	-	-	-	-	-	-	-	-	-
ωB97XD	6-311++G**	1.253	1.356	1.305	1.402	1.364	1.750	1.428	122.7	−77.0	171.2	−44.0
	Aug-cc-pvdz	-	-	-	-	-	-	-	-	-	-	-
Expert. ^1^		1.301	1.351	1.311	1.421	1.361	1.738	-	-	80	-	−63

^1^ Taken from Ref. [31]. Atom numbering is according to that of Figure 1.

**Table 2:** Absolute and average absolute errors of some selected bond lengths (in Å) and torsional angles (in deg.) for the thiazole azo dyes B (top 7 lines) and C (bottom 7 lines), which were estimated by using different DFT functionals with 6-311++G** and aug-cc-pvdz basis sets as compared to the experimental crystal data ^1^. The crystal structure of A is not available.

Parameter		N6–N7	N5–C2	N5–C3	C1–C2	C33–C50	S4–C1	S4–C3	C15–N18–C26–C29	C15–N18–C19–C22	C41–C40–C2–N5
B3LYP	6-311++G**	0.005	0.010	0.007	0.026	0.013	0.015	0.033	5.6	8.4	2.4
	Aug-cc-pvdz	0.020	0.006	0.002	0.030	0.017	0.020	0.037	5.7	8.4	1.9
CAM-B3LYP	6-311++G**	0.043	0.009	0.018	0.009	0.003	0.002	0.010	7.5	8.7	4.0
	Aug-cc-pvdz	0.038	0.006	0.014	0.014	0.002	0.008	0.015	7.5	8.7	3.5
ωB97XD	6-311++G**	0.043	0.008	0.017	0.010	0.002	0.000	0.010	12.8	8.1	5.0
	Aug-cc-pvdz	0.038	0.005	0.012	0.014	0.003	0.006	0.015	12.8	8.0	4.7
Average		0.031	0.007	0.012	0.017	0.007	0.009	0.020	8.7	8.4	3.6
B3LYP	6-311++G**	0.029	0.002	0.005	0.002	0.020	0.029	0.025	2.3	0.6	23.7
	Aug-cc-pvdz	0.025	0.005	0.009	0.006	0.026	0.035	0.030	2.4	0.5	25.2
CAM-B3LYP	6-311++G**	0.048	0.003	0.008	0.018	0.004	0.015	0.004	1.2	0.1	21.4
	Aug-cc-pvdz	-	-	-	-	-	-	-	-	-	-
ωB97XD	6-311++G**	0.048	0.005	0.006	0.019	0.003	0.012	0.006	3.0	1.1	19
	Aug-cc-pvdz	-	-	-	-	-	-	-	-	-	-
Average		0.038	0.004	0.007	0.011	0.013	0.023	0.016	2.2	0.6	22.3

^1^ Taken from Ref. [31]. Atom numbering is according to that of Figure 1.

**Table 3:** The HOMO (highest occupied molecular orbital) and LUMO (lowest unoccupied molecular orbital) orbitals energies (eV), the energy gaps (E.G./eV), the dipole moments (D.M./Debye) the chemical hardness (η/eV), electronic chemical potential (µ/eV), the global electrophilicity index (ω/eV) and the total hyperpolarizabilities (β_tot_/au) for gas-phase dyes A, B and C, which were calculated by applying B3LYP, CAM-B3LYP and ωB97XD functionals with 6-311++G** and aug-cc-pvdz basis sets. For comparison, the values for gas-phase p-nitroaniline (pNA) are given.

Dye	Parameter	B3LYP		CAM-B3LYP		ωB97XD	
		6-311++G**	aug-cc-pvdz	6-311++G**	aug-cc-pvdz	6-311++G**	aug-cc-pvdz
A	HOMO	−5.541	−5.503	−6.755	−6.708	−7.261	−7.220
	LUMO	−3.115	−3.103	−2.040	−2.030	−1.459	−1.457
	E.G.	2.426	2.400	4.715	4.678	5.802	5.763
	D.M.	8.66	8.72	7.03	7.13	6.77	6.88
	η	1.213	1.200	2.358	2.339	2.901	2.882
	μ	4.328	4.303	4.398	4.369	4.360	4.339
	ω	7.721	7.715	4.101	4.080	3.276	3.266
	β_tot_	24,750	24,596	19,938	20,324	17,363	18,326
B	HOMO	−5.826	−5.786	−7.035	−6.989	−7.536	−7.497
	LUMO	−3.386	−3.367	−2.320	−2.301	−1.742	−1.731
	E.G.	2.440	2.419	4.715	4.688	5.794	5.766
	D.M.	14.26	14.27	12.55	12.65	12.30	12.43
	η	1.220	1.210	2.358	2.344	2.897	2.883
	μ	4.606	4.577	4.678	4.645	4.639	4.614
	ω	8.695	8.657	4.640	4.602	3.714	3.692
	β_tot_	17,714	17,500	18,258	18,158	16,904	16,927
C	HOMO	−5.677	−5.638	−6.865	−6.824	−7.375	-
	LUMO	−3.442	−3.427	−2.427	−2.371	−1.852	-
	E.G.	2.235	2.211	4.438	4.453	5.523	-
	D.M.	14.05	14.11	11.92	11.76	11.65	-
	η	1.118	1.106	2.219	2.227	2.762	-
	μ	4.560	4.533	4.646	4.598	4.614	-
	ω	9.299	9.289	4.864	4.747	3.854	-
	β_tot_	39,756	39,063	34,274	30,424	27,619	-
pNA	D.M.	7.17	-	7.23	-	7.16	-
	β_tot_	1327	-	1350	-	1350	-
	Expert. ^a^ β	1072 ± 44					

^a^ Taken from Ref. [39].

**Table 4:** The Dipole moments (D.M./Debye), the energy gaps (E.G./eV), the total hyperpolarizabilities (β_tot_ × 10^−28^/esu) and the maximum absorption wavelength (λ_max_/nm) for the chloroform-solvated dyes (A, B and C) which were estimated by utilizing B3LYP, CAM-B3LYP and ωB97XD functionals with 6-311++G** and aug-cc-pvdz basis sets. Some experimental related values of the three dyes in chloroform are also listed for comparison purposes.

Level of Theory	Parameter	A	B	C
B3LYP/6-311++G**	D.M.	12.78	19.38	19.41
	E.G.	2.290	2.301	2.139
	β_tot_	5.9890	4.4821	9.7969
	λ_max_	605	587	659
B3LYP/aug-cc-pvdz	D.M.	12.91	19.52	-
	E.G.	2.269	2.286	-
	β_tot_	6.0588	4.4632	-
	λ_max_	612	593	-
CAM-B3LYP/6-311++G**	D.M.	9.76	16.19	15.53
	E.G.	4.515	4.496	4.298
	β_tot_	4.5591	4.4724	6.8587
	λ_max_	497	498	528
CAM-B3LYP/aug-cc-pvdz	D.M.	9.97	16.46	15.85
	E.G.	4.481	4.470	4.262
	β_tot_	4.7470	4.5521	7.1861
	λ_max_	505	504	539
ωB97XD/6-311++G**	D.M.	9.29	15.72	15.08
	E.G.	5.598	5.569	5.373
	β_tot_	4.0510	4.1197	5.9315
	λ_max_	484	487	511
ωB97XD/aug-cc-pvdz	D.M.	9.54	16.08	-
	E.G.	5.559	5.539	-
	β_tot_	4.2482	4.2476	-
	λ_max_	492	494	-
Expert. ^a^	λ_max_ (Expert)	623	619	686
	μβ_O_ × 10^−48^/esu)	740	800	1970

^a^ Ref. [31].

**Table 5:** The Dipole moments (D.M./Debye), the energy gaps (E.G./eV), the maximum absorption wavelength (λ_max_/nm), the maximum excitation energy (E_max_/eV) and the total hyperpolarizabilities (β_tot_ × 10^−28^/esu) for the CCl_4_, CHCl_3_, CH_2_Cl_2_ and DMSO solvated dyes (A, B and C) which were estimated by utilizing CAM-B3LYP functional with 6-311++G** basis sets.

Solvent	Parameter	A	B	C
CCl_4_	D.M.	8.971	15.120	14.469
	E.G.	4.577	4.557	4.345
	λ_max_	490.50	491.12	523.50
	E_max_	2.528	2.525	2.369
	β_tot_	3.2178	3.0893	4.9493
CHCl_3_	D.M.	9.761	16.186	15.530
	E.G.	4.515	4.496	4.298
	λ_max_	496.90	497.92	528.02
	E_max_	2.495	2.490	2.349
	β_tot_	4.5591	4.4724	6.8587
CH_2_Cl_2_	D.M.	10.160	16.706	16.052
	E.G.	4.483	4.465	4.274
	λ_max_	499.60	500.70	529.80
	E_max_	2.482	2.476	2.340
	β_tot_	5.4167	5.3578	8.0403
DMSO	D.M.	10.564	17.222	16.570
	E.G.	4.451	4.434	4.250
	λ_max_	503.10	504.10	532.20
	E_max_	2.465	2.460	2.330
	β_tot_	6.4435	6.4101	9.4206

**Table 6:** Some selected most efficacious second-order perturbation ($E^{\left(2\right)}$) assessment of the hyperconjugative energies (kcal/mol) that trace the charge transfer from the methoxyphenyl donor group to the indandione, dicyanovinyl and dicyanovinylindanone acceptor moieties of the thiazole azo dyes A, B and C, respectively. They were computed applying HF/6-31+G*//B3LYP/6311++G** level of theory.

Interaction	A	B	C
n_2O45_→π*_C38–C42_	44.70	45.65	44.95
π_C38–C42_→π*_C35–C36_	58.80	54.83	55.10
π_C38–C42_→π*_C37–C40_	30.69	30.83	30.08
π_C35–C36_→π*_C1–C2_	23.31	23.69	22.83
π_C1–C2_→π*_C35–C50_	40.80	43.29	49.30
π_C33–C50_→π*_C51–C63_	-	-	37.99
π_C33–C50_→π*_C51–O64_	34.05	-	-
π_C33–C50_→π*_C52–O63_	33.30	-	31.75
π_C33–C50_→π*_C51–N53_	-	30.50	30.79
π_C33–C50_→π*_C52–N54_	-	28.67	29.99
Total	265.65	257.46	332.78
